# Analysis of laboratory markers in body contouring procedures after bariatric surgery does not indicate particular risks for perioperative complications

**DOI:** 10.1186/s13741-024-00422-7

**Published:** 2024-06-27

**Authors:** Maximilian C. Stumpfe, Juliane Platzer, Raymund E. Horch, Alexander Geierlehner, Andreas Arkudas, Wibke Mueller-Seubert, Aijia Cai, Theresa Promny, Ingo Ludolph

**Affiliations:** Department of Plastic and Hand Surgery and Laboratory for Tissue Engineering and Regenerative Medicine, University Hospital Erlangen, Friedrich Alexander University Erlangen-Nürnberg FAU, Krankenhausstraße 12, Erlangen, 91054 Germany

**Keywords:** Body contouring, Bariatric surgery, Laboratory markers, Complication, Obesity, Massive weight loss

## Abstract

**Background:**

Body contouring surgery after massive weight loss is associated with different risk factors. Wound healing disorders and seromas commonly occur postoperatively. Bariatric interventions lead to massive weight loss with excess skin and soft tissue. In this study, perioperatively collected laboratory markers of this special patient population were analyzed.

**Methods:**

Fifty-nine patients were analyzed retrospectively regarding bariatric surgery, weight loss, body contouring surgery, laboratory markers, and complication rates.

**Results:**

Body contouring surgery (*n* = 117) was performed in 59 patients. Weight loss was achieved after gastric bypass (40.1%), gastric banding (33.9%), or sleeve gastrectomy (26.0%), with an average of 69.2 kg. The most common body contouring procedure included abdominoplasty (*n* = 50), followed by thigh lift (*n* = 29), mammaplasty (*n* = 19), brachioplasty (*n* = 14), and upper body lift (*n* = 5). Analysis of laboratory markers revealed no exceptional and clinically relevant variations. Correlation analysis revealed associations between resection weight, amount of drain fluid, and particular laboratory markers.

**Conclusion:**

Analysis of perioperative laboratory markers in this special patient population after massive weight loss did not indicate clinically relevant risk factors regardless of the type of bariatric or body contouring surgery. Body contouring surgeries after bariatric interventions prove to be safe and low risk concerning perioperative laboratory markers and postoperative hospitalization.

## Introduction

After massive weight loss (MWL), patients are afflicted with special conditions such as excess skin tissue. The resulting difficulties have already been discussed prior to this study (Cai et al. 2020). However, before MWL, patients usually suffer from multimorbidity. Patients with obesity are 5.5–6.0 times more likely to experience metabolic syndrome than normal-weight patients (Engin 2017). Metabolic syndrome comprises abdominal obesity, dyslipidemia, hyperglycemia, and hypertension and is a major public health challenge. Abdominal obesity is the most frequently identified metabolic condition (Eckel et al. 2005). Commonly, this comes along with alterations in specific laboratory markers associated with a metabolic syndrome. Patients undergoing massive weight loss additionally showed a reduction in metabolic syndrome disorders (Nguyen and Varela 2017; Schlottmann et al. 2018). Nevertheless, bariatric surgical approaches can lead to or intensify nutrient deficiency (Bal et al. 2012). Micronutrient supplementation is crucial to the follow-up period of bariatric surgery (Tabesh et al. 2019). Despite evidence of long-term weight loss and remission of obesity-related comorbidities, patients undergoing body contouring surgery continue to have special risks and requirements. Various patients are still overweight due to stagnating or insufficient weight loss (Hauck et al. 2019). Despite improvements in obesity-related diseases, not all patients have complete remission (Nguyen and Varela 2017). Body contouring surgeries are considered safe procedures. However, common perioperative complications, such as seroma, wound healing disorders, and bleeding, remain (Hauck et al. 2019; Michaels et al. 2011). Wound healing depends on multiple macro- and micronutrients. Protein deficits lead to an increased inflammatory response (Romano et al. 2019). Additionally, vitamins A/C/D/B12, folate, thiamin, iron, ferritin, zinc, and selenium are described as essential elements of wound healing (Agha-Mohammadi and Hurwitz 2010). The compensation of these deficits was shown to decrease those complications (Agha-Mohammadi and Hurwitz 2010). Barbour et al., on the other hand, saw no effect of the substitution of the previously mentioned nutrients on the complication rate of panniculectomy (Barbour et al. 2015). However, common laboratory markers (e.g., hemoglobin, CRP, leukocytes) have not yet been assessed perioperatively as a prognostic value for this group of patients. Therefore, in this study, we investigated specific laboratory markers and complications in the perioperative course of surgical body contouring interventions in this special patient collective. We addressed the question of how laboratory markers are related to different complications and whether one can reduce possible risk factors to further reduce perioperative complications.

## Materials and methods

In a single-center study, we retrospectively reviewed 59 patients undergoing bariatric (gastric banding, gastric sleeve, gastric bypass) and multistage body contouring surgery treated exclusively in our interdisciplinary obesity center. We excluded patients with dietary weight loss and patients who received bariatric surgery in another hospital.

Body contouring surgery included abdominoplasty, thigh lift, brachioplasty, upper body lift, and mammaplasty. Outpatient records, clinical documentation programs, and nursing documentation were screened for the parameters shown in Table 1.

**Table 1:** Collected data of the patient collective

Patient demographics	Age, gender, secondary diagnosis
Bariatric surgery	BMI before surgery, weight loss procedure, weight loss
Body contouring surgery	BMI before surgery, the type of body contouring, resection weight, duration of hospitalization
Wound healing Outcomes	Bleeding, wound infection, wound dehiscence according to the Clavien‒Dindo-classification, amount of drained fluid

All included data were managed on a bespoke Excel sheet (Microsoft, Redmond, WA, USA).

### Operative procedure

#### Bariatric intervention

Patients underwent bariatric intervention through the Department of General and Visceral Surgery as a partner of the obesity center. This involved the application of a gastric band, a gastric bypass, or a gastric sleeve in our study group. Follow-up postoperative laboratory measurements were performed regularly (see the “Perioperative blood sampling” section). Patients received standardized supplementation of relevant micronutrients and vitamins depending on the operative procedure.

#### Body contouring surgery

We performed abdominoplasty, thigh lifts, mammaplasty, brachioplasty, and upper body lifts in a multistage concept. Surgical drains were routinely used in all procedures. During hospitalization, the patients received either low-molecular-weight heparin or unfractionated heparin. Antibiotics, usually cephalosporins, were administered intravenously intra- and postoperatively. There was no substitution of additional vitamins and micronutrients perioperatively.

### Perioperative blood sampling

Laboratory markers from the obesity center were available for this study depending on the bariatric procedure. Blood sampling was performed at 3 (gastric bypass), 6 (gastric sleeve and gastric bypass), and 12 months (gastric sleeve and gastric bypass) in the first year. After the first postoperative year, laboratory markers were repeated every 6–12 months to control nutritional deficiencies.

According to our routine, blood sampling was usually performed on the day before the body contouring surgery and on the first postoperative day. Further laboratory tests were performed depending on the individual postoperative course. We recorded the preoperative values as well as the last blood test before discharge of the patients from the hospital. Furthermore, we evaluated postoperative laboratory control in the long-term course depending on the above-described interval. To summarize the laboratory values in the first postoperative days, we evaluated the differences between the preoperative value and the maximum increase or decrease in the postoperative course. Attention was given to the laboratory markers that could influence an operation or vice versa. The analyzed values can be taken from Table 2.

**Table 2:** Retrospective data evaluation of the mentioned laboratory markers

General laboratory markers	Hemoglobin, thrombocytes, leukocytes, c-reactive protein, creatinine, electrolytes
Specific laboratory markers	Magnesium, folic acid, protein, albumin, iron, ferritin, parathormone, HbA1c, vitamin D3, vitamin B1, vitamin B2, vitamin B6, vitamin B12

### Statistical analysis

We analyzed patient demographics and the subgroup analysis by descriptive statistics. Descriptive statistics are expressed as the mean ± standard deviation (SD) for parametric data and as the median ± standard deviation (SD) for nonparametric data. A paired sample *t*-test or Wilcoxon’s signed-rank test was used for significance tests. Significance was set at a *p*-value < 0.05. Correlation analysis was performed using Pearson correlation with a confidence interval of 95%. The correlation coefficient was set according to Cohen (effect size small: 0.01–0.3; effect size medium: 0.3–0.5; effect size large: higher 0.5) (Cohen 1988).

Analyses were performed using GraphPad Prism Version 9 (GraphPad Software, Inc., CA, USA).

### Ethical approval

The study is in accordance with the 1964 Helsinki Declaration and its later amendments and comparable ethical standards. The paper is exempt from ethical committee approval. This is a retrospective study, and the paper does not report on primary research. All data analyzed were collected as part of routine diagnosis and treatment. These data were anonymized.

## Results

### Total patient collective demographics

**Fig. 1:**
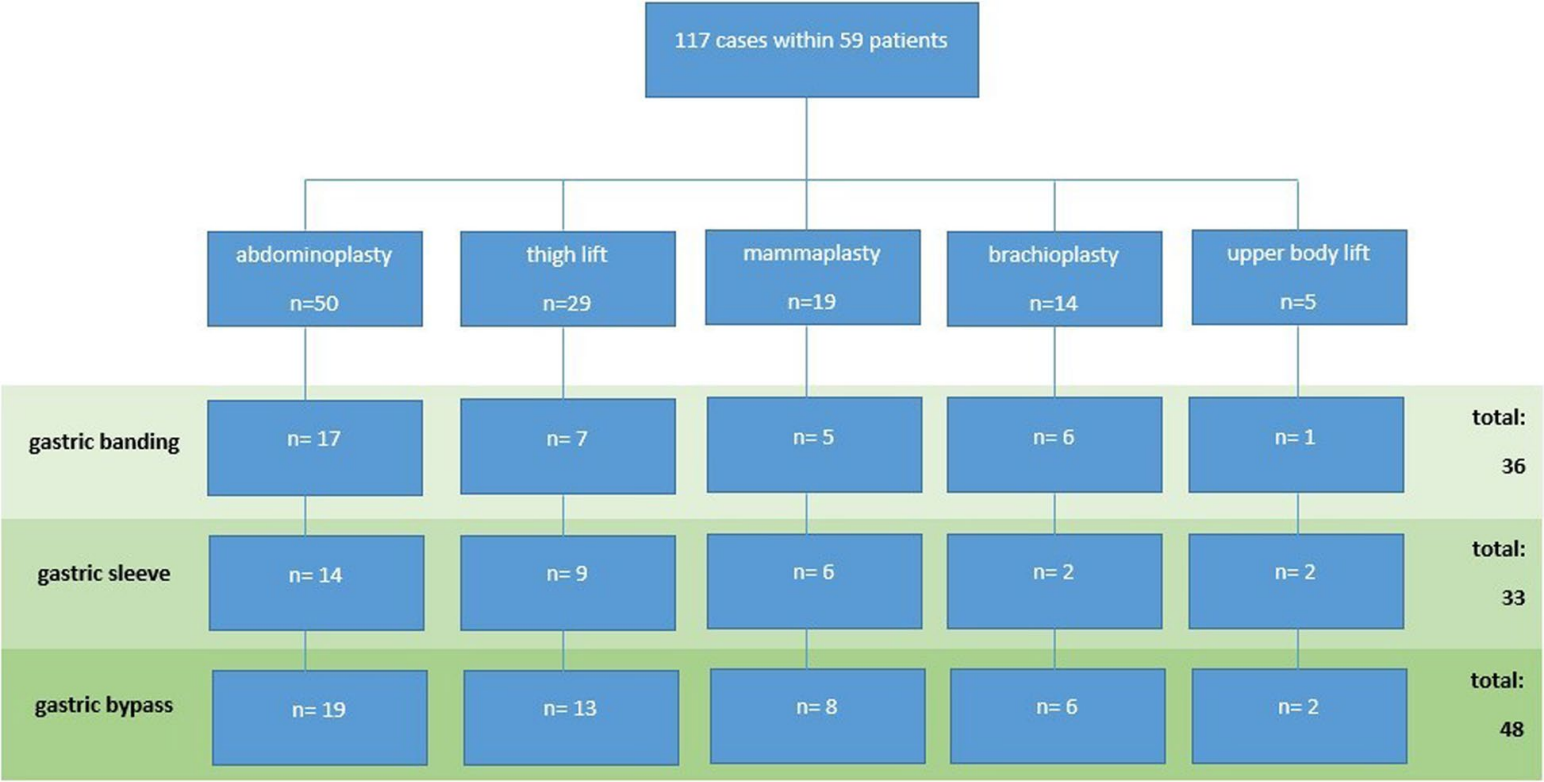
Presentation of cases with segmentation into body contouring surgeries as well as breakdown of bariatric procedures for each plastic surgery

We included 59 patients (17 males/42 females) with a total of 117 body contouring surgeries (Fig. 1). The mean age was 43.5 years (range 20–60 years). Weight loss was achieved by gastric bypass (24 patients/40.1%), gastric banding (20 patients/33.9%), or gastric sleeve (15 patients/26.0%). Two patients received conversion surgery to gastric bypass prior to their first body contouring surgery.

The overall average weight loss was 69.2 kg (range 29–150 kg). The mean BMI before body contouring surgery was 30.4 kg/m^2^ (range 20.4–53.0 kg/m^2^). The mean resection weight of all surgeries was 1666 g (range 122–6015 g). The most frequently performed body contouring surgery was abdominoplasty (50 cases). Thigh lifts were performed in 29 cases and mammaplasty in 19 cases. Brachioplasty and upper body lifts amounted to 14 cases and 5 cases, respectively.

### Bariatric procedure demographics

The most commonly performed bariatric procedure was gastric bypass. The highest weight loss was recorded in the group of patients with sleeve gastrectomy. Similarly, these patients had the highest resection weight during body contouring procedures. Table 3 includes descriptive information about the bariatric subgroups.

**Table 3:** Descriptive data of patients after bariatric procedures

Characteristic	Gastric banding	Gastric sleeve	Gastric bypass
*Number of body contouring surgeries*	36	33	48
*Female/male, no. (%)*	28 (78%)/8 (22%)	16 (48%)/17 (52%)	43 (89%)/5 (11%)
*Age at body contouring procedure (mean, range) (years)*	42.4 (23–58)	41.64 (28–50)	45.7 (20–60)
*BMI (median, range) (kg/m*^*2*^*)*	30.8 (23.5–53.0)	31.6 (22.7–39.5)	29.2 (20.4–46.1)
*Total weight loss (mean, range) (%)*	37.7 (24.8–52.8)	45.1 (30.2–58.7)	45.0 (25.9–60.0)
*Resection weight (mean, range) (g)*	1419 (180–3840)	1966 (122–5000)	1630 (175–6015)
*Total amount of drained fluid during hospitalization of the body contouring procedure (mean, range) (ml)*	980 (90–3475)	1279 (220–4160)	1319 (55–7395)
*Duration of hospitalization of the body contouring procedure (mean, range) (days)*	7.9 (3–21)	6.7 (3–13)	8.3 (3–21)

### Perioperative laboratory controls

#### Standard laboratory values

Preoperatively, the mean values of hemoglobin (mean 13.3 g/dl; range 8.5–16.9 g/dl), thrombocytes (mean 268.5 × 103/µl; range 148–702 × 103/µl), leukocytes (mean 7 × 103/µl; range 3.6–13.8 × 103/µl), CRP (mean 2.3 mg/l; range 0.2–13.3 mg/l), and creatinine (mean 0.8 mg/dl; range 0.5–1.4 mg/dl) presented within the reference range for all bariatric and postbariatric procedures. When considering the laboratory values shown in Table 4, only the postoperative hemoglobin and CRP showed alterations beyond the normal range. A closer look at the hemoglobin revealed a significant decrease from the preoperative value to the postoperative laboratory control before discharge. This statistically significant difference for the hemoglobin marker was most evident in thigh lifts regardless of the bariatric procedure (gastric banding: *p* = 0.0047, gastric sleeve: *p* = 0.0206, and gastric bypass: *p* = 0.0057). The long-term follow-up blood control was taken on average approximately 2 months after body contouring surgery. No significant variances from the baseline values before body contouring surgery were observed. One patient received red blood cell transfusions; therefore, these data were not considered for the analysis. Including patients with postoperative bleeding (according to Clavien‒Dindo I and IIIb) in the total study cohort, the mean hemoglobin values were 11.2 g/dl (Δ-2.5 g/dl; gastric banding), 11.6 g/dl (Δ-2.5 g/dl; gastric sleeve), and 10.5 g/dl (Δ-2.6 g/dl; gastric bypass). In comparison, in the collective excluding patients with postoperative bleeding (7 patients were excluded; see Table 6), a postoperative mean of 11.3 g/dl (Δ-2.4 g/dl; gastric banding), 11.8 g/dl (Δ-2.4 g/dl; gastric sleeve), and 10.6 g/dl (Δ-2.5 g/dl; gastric bypass) was observed.

**Table 4:** Laboratory markers with range according to the clinic laboratory — hemoglobin, thrombocytes, leukocytes, c-reactive protein/CRP, and creatinine — preoperatively, post-op (last laboratory control before discharge) and during the course (approx. 2 months). Also, the presentation of the maximum change (leukocytes, CRP, creatinine, hemoglobin, thrombocytes). The mean value is shown. A subdivision was made according to the body contouring and the bariatric surgery used

	**Abdominoplasty** **Total**	**Thigh lift** **Total**	**Mammaplasty** **Total**	**Brachioplasty** **Total**	**Upper body lift** **Total**
***Surgeries***	***50***	***29***	***19***	***14***	***5***
Hemoglobin-preop (g/dl) (12–15)	13.7	12.9	13.1	12.5	13.5
ΔHemoglobin (g/dl)	− 3.1	− 2.5	− 1.9	− 1.7	− 0.7
Hemoglobin-post-op (g/dl)	11.2	10.7	11.5	10.9	10.8
Hemoglobin-long term (g/dl)	12.3	13.3	11.3	12.9	13.3
Thrombocytes-preop (× 10^3^/µl) (160–400)	264.1	281.0	272.3	258.1	253.2
Δthrombocytes (× 10^3^/µl)	− 48.7	− 58.7	− 36.0	− 30.4	− 35.4
Thrombocytes-post-op (× 10^3^/µl)	289.2	273.5	244.4	244.9	233.2
Thrombocytes-long term (× 10^3^/µl)	288.5	263.3	306.8	269.2	270.5
Leukocytes-preop (× 10^3^/µl) (4–11.5)	6.8	7.3	7.1	7.3	6.6
Δleukocytes (× 10^3^/µl)	2.4	1.7	1.1	0.8	1.1
Leukocytes-post-op (× 10^3^/µl)	7.3	6.9	8.9	7.6	6.1
Leukocytes-course (× 10^3^/µl)	6.4	7.1	7.3	6.8	8.1
CRP-preop (mg/l) (− < 5)	2.9	1.9	2.5	1.6	1.0
ΔCRP (mg/l)	23.8	14.6	37.4	30.3	20.7
CRP-post-op (mg/l)	27.9	26.8	29.7	16.6	18.8
CRP-long term (mg/l)	5.6	4.4	2.2	2.1	0.5
Creatinine-preop (mg/dl) (0.51–0.95)	0.8	0.8	0.8	0.8	0.8
Δcreatinine (mg/dl)	0.1	0.1	0.1	0.1	0.1
Creatinine-post-op (mg/dl)	0.7	0.7	0.7	0.7	0.6
Creatinine-long term (mg/dl)	0.7	0.7	0.7	0.7	0.7

*Δ*Differences between the preoperative value and the maximum increase or decrease

Further details for standard laboratory values are shown in Table 4.

#### Nutritional status after bariatric surgery

The preoperative mean values of the necessary nutritional supplements following bariatric surgery were within the recommended range. Postoperative laboratory control of nutritional status was performed approximately 2 months after surgery and presented normal mean values. The detailed results can be found in Table 5.

**Table 5:** Laboratory markers of nutritional status following bariatric surgery. The mean value is shown

	**Range according to the obesity center**	**Total**	**Gastric banding**	**Gastric sleeve**	**Gastric bypass**
**Vitamin D3 preop**	*30.0–70.0 ng/ml*	30.6 (5.2–64.0)	23.6 (13.0–36.7)	24.1 (5.2–47.0)	35.4 (6.3–64.0)
**Vitamin D3 post-op**		31.0 (7.0–62.5)	29.9^a^	27.8 (11.8–43.2)	33.3 (7.0–62.5)
**Vitamin B1 preop**	*28.0–85.0 µg/l*	73.3 (39.8–207.0)	74.3 (71–79)	73.1 (46.1–207.0)	73.4 (39.8–137.0)
**Vitamin B1 post-op**		69.4 (37.8–87.4)	70.9^a^	70.3 (37.8–195.7)	70.7 (39.8–125.2)
**Vitamin B2 preop**	*137.0*–*370.0 µg/l*	204.4 (89.2–222.8)	219.7 (166–267)	172.2 (89.2–229.0)	219.3 (127–312)
**Vitamin B2 post-op**		201.9 (143.0–156.0)	187^a^	183.5 (143–226)	212.9 (144–299)
**Vitamin B6 preop**	*5.0*–*30.0 ng/ml*	17.0 (1.9–62.4)	8.6 (3.4–12.0)	13.9 (1.9–36.7)	19.2 (6.2–62.4)
**Vitamin B6 post-op**		18.3 (3.4–53.4)	42.3^a^	14.1 (3.4–44.4)	19.9 (5.0–53.4)
**Vitamin B12 preop**	*211*–*911 pg/ml*	450.7 (156.7–1360.0)	328 (174.8–444.1)	509.4 (156.7–1082.0)	431.1 (176.5–1360.0)
**Vitamin B12 post-op**		470.2 (169.5–1597.0)	516.7^a^	509.6 (169.5–1597.0)	442.7 (176.5–1177.0)
**HbA1c preop**	*4.4*–*6.0%*	5.6 (4.6–8.5)	5.3^a^	5.2 (4.8–5.5)	5.8 (4.6–8.5)
**HbA1c post-op**		5.5 (4.4–8.5)	5.9^a^	5.1 (4.4–5.7)	5.8 (4.8–8.5)
**Ferritin preop**	*22.0*–*112.0 ng/ml*	56.9 (8–248)	Na	65.7 (8–248)	39.3 (14–69)
**Ferritin post-op**		Na	Na	Na	Na
**Iron preop**	*35.0*–*145.0 µg/dl*	70.6 (11–189)	58.3 (37–78)	79.6 (20–189)	67.0 (11–134)
**Iron post-op**		73.9 (11–203)	46^a^	97.9 (20–203)	60.3 (11–134)
**Folic acid preop**	*3.89*–*20.0 ng/ml*	12.3 (2.6–20.0)	5.8 (3.7–6.9)	10.1 (2.6–20.0)	13.9 (4.5–20.0)
**Folic acid post-op**		12.9 (3.6–20.0)	20^a^	10.6 (3.6–20.0)	14.1 (6.9–20.0)
**Parathormone pre-op**	*15.0*–*65.0 pg/ml*	49.8 (15.7–145.0)	37.3 (33.0–45.7)	46.5 (31.8–89.2)	53.3 (15.7–145.0)
**Parathormone post-op**		52.5 (23.4–109.4)	53^a^	51.9 (34.3–109.4)	52.8 (23.4–98.2)
**Protein preop**	*66.0*–*83.0 g/l*	70.5 (34.8–48.5)	74.2 (70.9–77.4)	69.8 (62.7–77.8)	70.6 (63.7–79.2)
**Protein post-op**		69.3 (61.7–79.2)	69.3^a^	69.7 (63.5–78.6)	69.1 (61.7–79.2)
**Albumine preop**	*35.0*–*55.0 g/l*	42.4 (34.8–48.5)	45.5 (44–47)	41.9 (36.2–48.3)	42.5 (34.8–48.5)
**Albumin post-op**		41.6 (34.8–48.5)	42.3^a^	41.6 (36.2–45.8)	41.6 (34.8–48.5)
**Magnesium preop**	*0.7*–*1.1 mmol/l*	0.8 (0.7–0.9)	0.8 (0.8–0.9)	0.8 (0.7–0.9)	0.9 (0.8–0.9)
**Magnesium post-op**		1.0 (0.7–9.0)	0.8^a^	0.8 (0.7–0.9)	1.2 (0.7–9.0)

*Post-op* long term, *na* not available

^a^only one patient

**Fig. 2:**
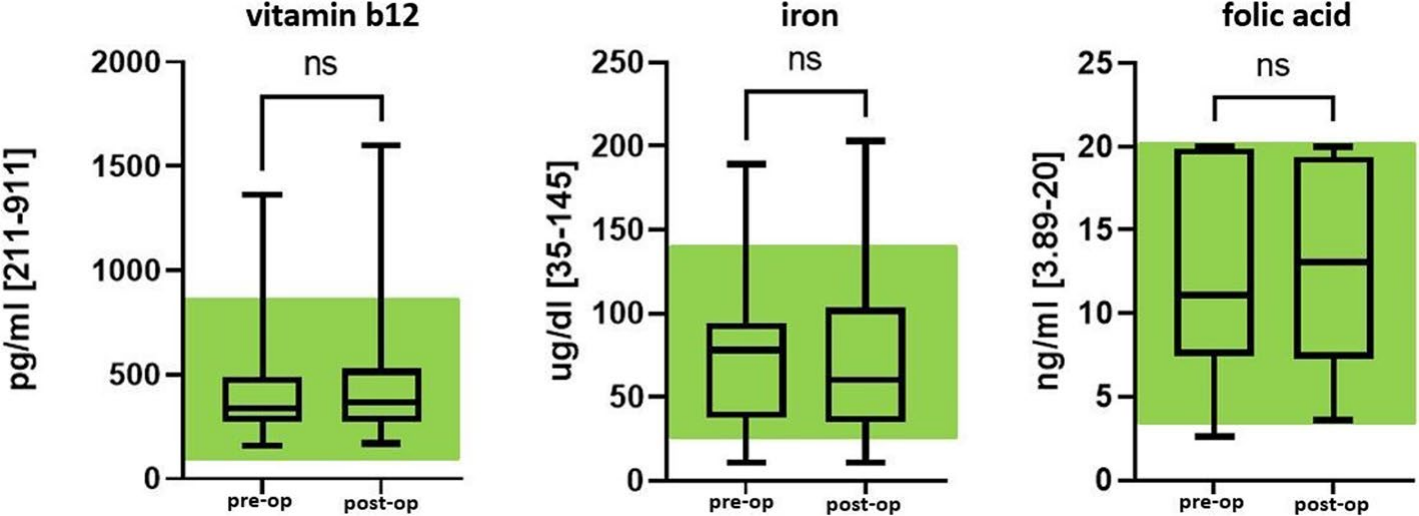
Important parameters (vitamin B12, iron, folic acid) for erythropoiesis preop and post-op (long term). Green area, normal range; *ns*, not significant

The necessary parameters for erythropoiesis are shown in Fig. 2.

### Complications after body contouring

In 13.7% of the cases (*n* = 15), there was an alteration from the regular postoperative course. In four cases (3.4%), an infection appeared, which was accompanied by an escalation of antibiotic therapy (according to Clavien‒Dindo grade II). Wound healing disorder occurred in four cases (3.4%) but did not require any intervention (according to Clavien‒Dindo grade I). Postoperative bleeding appeared in seven cases (4.6%), four of which needed surgical treatment (according to Clavien‒Dindo grade IIIb). One of those four patients received two red blood cell transfusions. A detailed overview with subdivisions of the bariatric and body contouring procedures can be obtained from Table 6.

**Table 6:** Complications of body contouring surgery

		**Infection**	**CD**	**Dehiscence**	**CD**	**Bleeding**	**CD**
**Gastric banding**	*Abdominoplasty*	0		2	I/I	1	IIIb
	*Thigh lift*	2	II/II	1	I	0	
	*Mammaplasty*	0		0		0	
	*Brachioplasty*	0		0		0	
	*Upper body lift*	0		0		0	
**Gastric sleeve**	*Abdominoplasty*	0		0		1	I
	*Thigh lift*	0		1	I	2	IIIb/IIIb
	*Mammaplasty*	1	II	0		0	
	*Brachioplasty*	0		0		0	
	*Upper body lift*	0		0		0	
**Gastric bypass**	*Abdominoplasty*	0		0		1	IIIb
	*Thigh lift*	0		0		0	
	*Mammaplasty*	1	II	0		1	I
	*Brachioplasty*	0		0		0	
	*Upper body lift*	0		0		1	I

*CD* Clavien‒Dindo

### Correlation between laboratory values and clinical parameters

A moderate correlation (*r* = 0.3–0.5) was observed between BMI before surgery and resection weight (*r* = 0.39; *p* < 0.0001). Likewise, a correlation between resection weight and the factors “amount of drain fluid” (*r* = − 0.39; *p* < 0.0001) and “Δhemoglobin” (*r* = − 0.33; *p* = 0.00011) could be detected. The amount of drain fluid correlated with “Δleukocytes” (*r* = − 0.30; *p* = 0.00015), “Δthrombocytes” (*r* = − 0.37; *p* < 0.0001), “Δhemoglobin” (*r* = − 0.38; *p* < 0.0001), and Δcreatinine (*r* = 0.31; *p* = 0.0026). Multiple small correlations (*r* < 0.3) for almost all other parameters were found.

## Discussion

Body contouring surgery after massive weight loss is associated with increased complication rates. Therefore, presurgical optimization of comorbidities and a check-up for surgery-related parameters to minimize postoperative complications are of high importance. The consensus among experts is to perform postbariatric surgery rather cautiously or even refuse to perform such procedures in patients who are still markedly obese (Hauck et al. 2019; Promny et al. 2021). The target weight and stability of body weight are important conditions for the decision upon an operative procedure. Moreover, interventions should be performed by experienced surgeons (Dragu and Horch 2014). A multistage concept is recommended in cases requiring contouring of various body parts to reduce postoperative complications.

As a consequence of massive weight loss, excessive skin and soft tissue remain, posing an often underestimated burden for the patient. Body contouring surgery can help to overcome chronic inflammatory skin disorders triggered by extensive skin-to-skin contact and psychological stress (Stumpfe et al. 2022; Baillot et al. 2013).

Health-related changes following bariatric interventions in obese patients are well documented in the literature. Metabolic diseases such as diabetes or hypertonia as well as skeletal disorders are positively influenced. Moreover, the optimization or even normalization of laboratory markers is known (Mohapatra et al. 2020). On the other hand, bariatric procedures can influence the absorption or metabolization of different nutrients with influence on laboratory markers necessitating routinely performed blood controls and lifelong substitution of vitamins and micronutrients.

Although bariatric surgery is currently a common procedure with an elevated risk of wound healing problems, to date, in postbariatric patients, there is a lack of knowledge about the influence of bariatric interventions on laboratory markers following body contouring procedures. The presented data in this study reveal no exceptional variances in the course of laboratory markers independent of the bariatric procedure or the body contouring intervention. Preoperative control, especially for surgery-related markers such as hemoglobin, leukocytes, thrombocytes, or creatinine, presented normal values. As expected, postoperative changes from the baseline in different values occurred, but there was no need for a medical intervention such as blood transfusion (except for one patient) or drug administration related to the variational value (except for five patients with infections). Even though values for hemoglobin were decreased and those for CRP were increased at the time of discharge from the hospital, those values presented a correction compared to the latest values measured during the hospital stay and were normalized by 2 months postoperatively. If hemoglobin levels are reduced preoperatively, Bayter-Martin et al. recommend parenteral iron and erythropoietin administration (Enrique Bayter-Marin et al. 2021). In contrast, the administration of iron to treat preoperative iron deficiency anemia was not considered useful by Ng et al. (Ng et al. 2015). Bleeding complications are common in this highly elective patient group. Risk should be reduced to a minimum, as bleeding complications lead not only to revisional surgery but also to more than three times more hospital readmissions (Vieira et al. 2017). Additionally, the long-term risk of secondary postoperative hematoma formation can occur (Dragu et al. 2009; Stumpfe et al. 2020). In elderly patients over 60 years of age, the risk for bleeding and hematoma was found to be increased (Fliss et al. 2022).

According to Correia-Sa and colleagues, a preoperatively elevated CRP value indicated an increased risk for the formation of hypertrophic scars (Correia-Sa et al. 2017). The study examined laboratory markers before surgery and during the course of 5 days afterwards. Patients who developed hypertrophic scars in the clinical follow-up 6 months postoperatively already had an elevated CRP value before surgery. Pathologic scarring is thought to be caused by a prolonged or increased inflammatory response (Niessen et al. 2004). For this reason, current prophylactic and treatment strategies (e.g., intralesional corticosteroid injection or adjuvant radiotherapy/brachytherapy) focus mainly on limiting inflammatory processes (Lee and Jang 2018). Long-term studies evaluating scar formation are necessary to underline or disprove this association.

In addition to the standard laboratory markers, a detailed evaluation of nutrients was performed in this study. Bariatric surgical approaches can cause or worsen nutrient deficiencies. Nutrient deficiency can lead to various health problems, including weakness, hematoma, and infections (Bal et al. 2012; Gruener et al. 2022). The existing guidelines form the basis for recommendations on supplementation and treatment after weight loss surgery (Stroh et al. 2017). Standardized approaches to micronutrient supplementation and clinical and laboratory screening for micronutrient deficiencies are needed after bariatric surgery. Lifelong supervision in an obesity center for bariatric surgery is mandatory (Mohapatra et al. 2020). In this context, care should be taken to avoid excessive intake of vitamin supplements, as this can lead to acute kidney failure and, over time, to chronic kidney failure (Francesco Daher et al. 2017). Patients with regular attendance at an obesity center as well as with high compliance showed sufficient medication supplementation. Our data showed that there is no increased risk for surgery due to a deficiency of micronutrients if the abovementioned steps have been carried out regularly. If the recommended laboratory controls are regularly performed, there are no abnormalities to be expected in the specific and general laboratory markers in bariatric surgery patients.

Bariatric weight loss surgery is generally associated with a higher risk for complications compared to dietary weight loss (Staalesen et al. 2012). However, in this study and in the literature, there is no evidence for an increased complication rate between the individual bariatric procedures (Garcia Botero et al. 2017; Pajula et al. 2019). A risk factor for wound healing problems is described by a higher resection weight and a preoperative BMI higher than 30 kg/m^2^ (Parvizi et al. 2015). Further risk factors are known and should be taken into account in the planning of surgical interventions in this patient group. Overall, the complication rate in our patient population was low. In centers with high experience in the field of bariatric surgery and body contouring procedures, those interventions can be performed with a high level of safety.

According to the data found in this study, the authors recommend that certain laboratory parameters should be consistently monitored. A general baseline measurement should be obtained, especially for the parameters hemoglobin, thrombocytes, leukocytes and CRP, and creatinine. If the hemoglobin value is noticeable, it should be controlled in advance or optimized, if necessary (Dragu and Horch 2014). As considered above, the administration of iron and erythropoietin for preoperative optimization of hemoglobin is controversial (Enrique Bayter-Marin et al. 2021; Ng et al. 2015). Due to the correlation analysis in cases of high resection weight and high seroma production as well as in cases of an increased risk of seroma formation, the abovementioned parameters hemoglobin, thrombocytes, leukocytes, and creatinine are recommended to be controlled in the postoperative course (Salari et al. 2021). The values mentioned should likewise be within the normal range preoperatively in order to be able to intercept complications in the event of a significant discrepancy (delta).

If there is no regular visit at an obesity center, patients are recommended to check values for protein, albumin, arginine, glutamine, vitamin a, vitamin b12, vitamin c, folate, thiamin, iron, zinc, and selenium before body contouring in accordance with the literature (Agha-Mohammadi and Hurwitz 2008). The healing of wounds and optimization of the immune system are influenced by these parameters (Hurwitz et al. 2008). Marouf et al. claim that a valuation of the prealbumin or transferrin level shows the patient’s current nutritional status the best (Marouf and Mortada 2021). However, Botero et al. did not find a correlation between preoperative serum albumin levels and the incidence of complications in patients with body contouring procedures (Garcia Botero et al. 2017).

This study is limited by its retrospective design. A further subdivision into detailed subgroups — with an already small subgroup size — is not meaningful and comparable due to the resulting group size. A dietary weight loss reference group would be a preferable addition but seems infeasible due to the lack of laboratory markers in the long term before and after body contouring procedures. Future studies could focus on factors influencing certain diets, such as vegetarian, vegan, alkaline, and ketogenic forms. These can have far-reaching effects on the laboratory parameters already considered and thus also on the results of the body contouring operation.

Considering the safety of body contouring procedures after bariatric interventions in view of surgery-related and specific laboratory markers, we could not find particular risks. The correlations found between an increased amount of drain fluid and the parameters hemoglobin, thrombocytes, leukocytes, and creatinine should be assessed in further studies including a larger patient population. The aforementioned correlations were of no clinical relevance in this study. Patients with more drained fluid had higher alterations in the aforementioned parameters; however, these alterations were not related to necessary treatment. Despite these results, perioperative monitoring of laboratory markers is essential, as well as frequent control by a specialized center. Comorbidities require special attention for related laboratory markers during the entire treatment. Similar to any other relevant surgical procedure, presurgical check-ups for variations in laboratory markers are important in the planning of surgical interventions. Hence, these are further relevant data to improve the safety of body contouring interventions and reduce complications.

## Conclusion

Among experts, it is well known that body contouring procedures are associated with certain complications. Because these procedures will still be frequently performed in the future, one should focus on further reducing common complications. Despite anatomical changes in the gastrointestinal tract, this analysis of perioperative laboratory parameters of the highly selective patient collective after massive weight loss does not indicate isolated specific clinically relevant risk factors independent of the type of bariatric procedure or weight reduction. In this special field of body contouring procedures, bariatric interventions causing metabolic changes seem not to influence the risk of laboratory changes perioperatively.

Postbariatric procedures in bariatric preoperated patients prove to be safe and low-risk options concerning the perioperative laboratory parameters and thus during postoperative hospitalization.

## Data Availability

The data presented in this study are available upon request from the corresponding author. The data are not publicly available due to the number of records.

## References

[CR1] Agha-Mohammadi S, Hurwitz DJ (2008). Nutritional deficiency of post-bariatric surgery body contouring patients: what every plastic surgeon should know. Plast Reconstr Surg..

[CR2] Agha-Mohammadi S, Hurwitz DJ (2010). Enhanced recovery after body-contouring surgery: reducing surgical complication rates by optimizing nutrition. Aesthetic Plast Surg..

[CR3] Baillot A, Asselin M, Comeau E, Meziat-Burdin A, Langlois MF (2013). Impact of excess skin from massive weight loss on the practice of physical activity in women. Obes Surg..

[CR4] Bal BS, Finelli FC, Shope TR, Koch TR (2012). Nutritional deficiencies after bariatric surgery. Nat Rev Endocrinol..

[CR5] Barbour JR, Iorio ML, Oh C, Tung TH, O'Neill PJ (2015). Predictive value of nutritional markers for wound healing complications in bariatric patients undergoing panniculectomy. Ann Plast Surg..

[CR6] Cai A, Maringa L, Hauck T, Boos AM, Schmitz M, Arkudas A (2020). Body contouring surgery improves physical activity in patients after massive weight loss-a retrospective study. Obes Surg..

[CR7] Cohen J (1988). Statistical Power Analysis for the Behavioral Sciences.

[CR8] Correia-Sa I, Serrao P, Marques M, Vieira-Coelho MA (2017). Hypertrophic scars: are vitamins and inflammatory biomarkers related with the pathophysiology of wound healing?. Obes Surg..

[CR9] De Francesco Daher E, Mesquita Martiniano LV, Lopes Lima LL, Viana Leite Filho NC, de Oliveira Souza LE, Duarte Fernandes PH (2017). Acute kidney injury due to excessive and prolonged intramuscular injection of veterinary supplements containing vitamins A, D and E: a series of 16 cases. Nefrologia..

[CR10] Dragu A, Horch RE (2014). [Concept of reconstructive body shaping in obesity] Evidence-based therapy algorithm. Chirurg.

[CR11] Dragu A, Bach AD, Polykandriotis E, Kneser U, Horch RE (2009). Pseudotumors after primary abdominal lipectomy as a new sequela in patients with abdominal apron. Obes Surg..

[CR12] Eckel RH, Grundy SM, Zimmet PZ (2005). The metabolic syndrome. Lancet..

[CR13] Engin A (2017). The definition and prevalence of obesity and metabolic syndrome. Adv Exp Med Biol..

[CR14] Enrique Bayter-Marin J, Cardenas-Camarena L, Pena WE, Duran H, Ramos-Gallardo G, Robles-Cervantes JA (2021). Patient blood management strategies to avoid transfusions in body contouring operations: controlled clinical trial. Plast Reconstr Surg..

[CR15] Fliss E, Manheim S, Zoabi T, Bashi T, Meilik B, Fliss-Isakov N (2022). The age factor in postbariatric body contouring surgery outcome. Plast Reconstr Surg..

[CR16] Garcia Botero A, Garcia Wenninger M, Fernandez Loaiza D (2017). Complications after body contouring surgery in postbariatric patients. Ann Plast Surg..

[CR17] Gruener JS, Horch RE, Geierlehner A, Mueller-Seubert W, Cai A, Arkudas A (2022). Is instillational topical negative pressure wound therapy in peri-prosthetic infections of the breast effective? A pilot study. J Pers Med..

[CR18] Hauck T, Schmitz M, Horch RE, Arkudas A, Boos AM, Cai A (2019). Operating on the edge? Body contouring procedures in patients with body mass index greater 35. Obes Surg..

[CR19] Hurwitz DJ, Agha-Mohammadi S, Ota K, Unadkat J (2008). A clinical review of total body lift surgery. Aesthet Surg J..

[CR20] Lee HJ, Jang YJ (2018). Recent understandings of biology, prophylaxis and treatment strategies for hypertrophic scars and keloids. Int J Mol Sci..

[CR21] Marouf A, Mortada H (2021). Complications of body contouring surgery in postbariatric patients: a systematic review and meta-analysis. Aesthetic Plast Surg..

[CR22] Michaels JT, Coon D, Rubin JP (2011). Complications in postbariatric body contouring: postoperative management and treatment. Plast Reconstr Surg..

[CR23] Mohapatra S, Gangadharan K, Pitchumoni CS (2020). Malnutrition in obesity before and after bariatric surgery. Dis Mon..

[CR24] Ng O, Keeler BD, Mishra A, Simpson A, Neal K, Brookes MJ (2015). Iron therapy for pre-operative anaemia. Cochrane Database Syst Rev..

[CR25] Nguyen NT, Varela JE (2017). Bariatric surgery for obesity and metabolic disorders: state of the art. Nat Rev Gastroenterol Hepatol..

[CR26] Niessen FB, Schalkwijk J, Vos H, Timens W (2004). Hypertrophic scar formation is associated with an increased number of epidermal Langerhans cells. J Pathol..

[CR27] Pajula S, Jyranki J, Tukiainen E, Koljonen V (2019). Complications after lower body contouring surgery due to massive weight loss unaffected by weight loss method. J Plast Reconstr Aesthet Surg..

[CR28] Parvizi D, Friedl H, Wurzer P, Kamolz L, Lebo P, Tuca A (2015). A multiple regression analysis of postoperative complications after body-contouring surgery: a retrospective analysis of 205 patients : regression analysis of complications. Obes Surg..

[CR29] Promny D, Hauck T, Cai A, Arkudas A, Heller K, Wullich B (2021). Abdominal panniculectomy can simplify kidney transplantation in obese patients. Urol Int..

[CR30] Romano L, Zoccali G, Orsini G, Giuliani M (2019). Reducing complications in post-bariatric plastic surgery: our experience and literature review. Acta Biomed..

[CR31] Salari N, Fatahi B, Bartina Y, Kazeminia M, Heydari M, Mohammadi M (2021). The global prevalence of seroma after abdominoplasty: a systematic review and meta-analysis. Aesthetic Plast Surg..

[CR32] Schlottmann F, Galvarini MM, Dreifuss NH, Laxague F, Buxhoeveden R, Gorodner V (2018). Metabolic effects of bariatric surgery. J Laparoendosc Adv Surg Tech A..

[CR33] Staalesen T, Olsen MF, Elander A (2012). Complications of abdominoplasty after weight loss as a result of bariatric surgery or dieting/postpregnancy. J Plast Surg Hand Surg..

[CR34] Stroh C, Manger T, Benedix F (2017). Metabolic surgery and nutritional deficiencies. Minerva Chir..

[CR35] Stumpfe MC, Horch RE, Geierlehner A, Ludolph I (2020). Rare pseudotumor-like hematoma at the latissimus dorsi muscle flap donor site: a treatment strategy utilizing negative pressure wound therapy with instillation and dwell time. Wounds..

[CR36] Stumpfe MC, Horch RE, Arkudas A, Cai A, Muller-Seubert W, Hauck T (2022). The value of negative-pressure wound therapy and flap surgery in hidradenitis suppurativa - a single center analysis of different treatment options. Front Surg..

[CR37] Tabesh MR, Maleklou F, Ejtehadi F, Alizadeh Z (2019). Nutrition, physical activity, and prescription of supplements in pre- and post-bariatric surgery patients: a practical guideline. Obes Surg..

[CR38] Vieira BL, Dorfman R, Turin S, Gutowski KA (2017). Rates and predictors of readmission following body contouring procedures: an analysis of 5100 patients from the National Surgical Quality Improvement Program Database. Aesthet Surg J..

